# Arsenic modifies the effect of folic acid in spina bifida prevention, a large hospital-based case-control study in Bangladesh

**DOI:** 10.21203/rs.3.rs-3989039/v1

**Published:** 2024-02-29

**Authors:** Chih-Fu Wei, Sudipta Kumar Mukherjee, Sheikh Muhammad Ekramullah, D. M. Arman, Md Joynul Islam, Mubinul Azim, Asifur Rahman, Md Nafaur Rahman, Md. Ziauddin, Gwen Tindula, Hafiza Sultana Suchanda, Diana F. Gomberg, Marc G. Weisskopf, Liming Liang, Benjamin C. Warf, David C. Christiani, Maitreyi Mazumdar

**Affiliations:** Harvard T.H. Chan School of Public Health; National Institute of Neurosciences & Hospital; National Institute of Neurosciences & Hospital; National Institute of Neurosciences & Hospital; National Institute of Neurosciences & Hospital; Dhaka Shishu Hospital; Bangabandhu Sheikh Mujib Medical University (BSMMU); National Institute of Neurosciences & Hospital; National Institute of Neurosciences & Hospital; Stanford University; National Institute of Neurosciences & Hospital; Boston Children’s Hospital; Harvard T.H. Chan School of Public Health; Harvard T.H. Chan School of Public Health; Boston Children’s Hospital; Harvard T.H. Chan School of Public Health; Harvard T.H. Chan School of Public Health

**Keywords:** Arsenic, Bangladesh, folic acid, spina bifida

## Abstract

**Background:**

Spina bifida, a developmental malformation of the spinal cord, is associated with high rates of mortality and disability. Although folic acid-based preventive strategies have been successful in reducing rates of spina bifida, some areas continue to be at higher risk because of chemical exposures. Bangladesh has high arsenic exposures through contaminated drinking water and high rates of spina bifida.

**Methods:**

We conducted a hospital-based case-control study at the National Institute of Neurosciences & Hospital (NINS&H) in Dhaka, Bangladesh, between December 2016 and December 2022. Cases were infants under age one year with spina bifida and further classified using data from observations by neurosurgeons and available imaging. Controls were drawn from children who presented to NINS&H or Dhaka Shishu Hospital (DSH) during the same study period. Mothers reported folic acid use during pregnancy, and we assessed folate status with serum assays. Arsenic exposure was estimated in drinking water using graphite furnace atomic absorption spectrophotometry (GF-AAS) and in toenails using inductively coupled plasma mass spectrometry (ICP-MS).

**Results:**

We evaluated data from 294 cases of spina bifida and 163 controls. We did not find a main effect of mother’s arsenic exposure on spina bifida risk. However, in stratified analyses, folic acid use was associated with lower odds of spina bifida (adjusted odds ratio [OR]: 0.50, 95% confidence interval [CI]: 0.25–1.00, p = 0.05) among women with toenail arsenic concentrations below the median, and no association was seen among mothers with toenail arsenic concentrations higher than median (adjusted OR: 1.09, 95% CI: 0.52–2.29, p = 0.82).

**Conclusions:**

Mother’s arsenic exposure modified the protective association of folic acid with spina bifida. Increased surveillance and additional preventive strategies, such as folic acid fortification and reduction of arsenic, are needed in areas of high arsenic exposure.

## BACKGROUND

Spina bifida is a developmental malformation of the spinal cord that leads to increased risk of mortality and disability, including leg, bladder, and bowel dysfunction, susceptibility to infection, hydrocephalus, and cerebrospinal fluid (CSF) leakage.[1] Spina bifida is a type of neural tube defect (NTD), a group of disorders caused by failure of the neural tube to fuse in the third week of gestation.[1] A recent analysis estimated that globally, at least 213,800 – 322,000 pregnancies are affected by NTDs each year, and in low-income and middle-income countries, the prevalence of NTDs exceeds one in every 100 births.[2]

Folic acid supplement use reduces the risk of spina bifida,[3] and fortification of staple foods with folic acid has been successful in decreasing spina bifida rates in multiple countries.[4, 5] However, a substantial number of affected pregnancies occur in areas with folic acid fortification and to women known to have taken folic acid supplements,[6] and the effectiveness of folic-acid based preventive strategies varies significantly across and even within countries.[2, 5] There is an urgent need to identify modifiable factors that may reduce the burden of this condition.

Environmental exposure to arsenic is of particular concern and may be a potential contributor to spina bifida risk, especially in areas of the world with high arsenic exposures and micronutrient deficiencies. Arsenic induces neural tube defects in several animal models,[7–11] and arsenic toxicity is closely related to one-carbon metabolism nutrients including folate.[12] A recent systematic review showed there was inadequate evidence to determine the relationship between prenatal arsenic exposure and prevalence of NTDs,[13] but the review did not address potential interactions between arsenic and folic acid that may affect spina bifida risk.

Understanding the relationship between folic acid, spina bifida, and arsenic exposure is especially important in Bangladesh, where an estimated 70 million people are chronically exposed to high concentrations of arsenic through contaminated groundwater, in what has been described as the largest mass poisoning in history.[14, 15] In 2019, a national survey conducted by the Bangladesh Bureau of Statistics and United Nations Children’s Fund estimated that 18.6% of households in Bangladesh were exposed to source water arsenic levels > 10 μg/L and 11.8% were exposed to levels > 50 μg/L.[16] Bangladesh also has high rates of spina bifida. The country does not have systematic surveillance for birth defects, but large hospital-based studies estimate the prevalence of spina bifida to be between 10.4 and 38.2 per 10,000 births.[17, 18]

Our previous study discovered that higher water arsenic concentrations reduced the effectiveness of folic acid in spina bifida prevention.[19] Based on these findings, we used toenail arsenic as biomarker, and identified an association between father’s arsenic exposure and spina bifida.[20] In this study, we sought to ascertain whether environmental arsenic exposure is associated with higher risk of spina bifida and whether arsenic exposure modified the protective effect of folic acid supplementation in Bangladesh.

## METHODS

### Case ascertainment, and control selection

We conducted a hospital-based case-control study at the National Institute of Neurosciences & Hospital (NINS&H), the primary center for spina bifida surgery in Bangladesh. Cases were infants with spina bifida who were less than one year old and had mothers who could identify their primary drinking water source during early pregnancy. Neurosurgeons at NINS&H examined all cases and reviewed medical records, operative reports, and available imaging results. Study staff recorded the subtype of spina bifida (myelomeningocele or meningocele), level of lesion (e.g., cervical, thoracic, lumbar, sacral, or lumbosacral), co-occurring anomalies, and complications present at time of enrollment, including cerebrospinal fluid leak, lesion infection, and hydrocephalus. A senior neurosurgeon (BCW) confirmed classification of cases by reviewing photographs and available imaging.

We selected controls from children who presented to NINS&H or the adjacent Dhaka Shishu Hospital (DSH), a children’s hospital immediately adjacent to NINS&H with similar referral patterns. Potential controls were seen for conditions including craniosynostosis, arachnoid cyst, trauma, subdural effusion, and epilepsy. Children with brain tumors or other cancers were not eligible to be controls. When a case was enrolled, we reviewed that week’s clinic lists and identified potential controls who were within six months of age of the enrolled case. The Bangladesh Medical Research Council and the Human Research Committees at Boston Children’s Hospital (BCH), NINS&H, and DSH approved this study (BCH protocol number IRB-P00019768; BMRC registration number: 006 23 08 2016). The Harvard T.H. Chan School of Public Health ceded review to BCH (protocol number: IRB20–0780). Parents provided informed consent before enrollment.

### Clinical information and folate status

Trained study staff interviewed patients’ families to collect demographic characteristics, patient and family medical histories, referral patterns to NINS&H, and medical histories. We measured infant’s weight and head circumference using standardized protocols, and mother’s medication and vitamin intake using a structured questionnaire that included the seven major types of folic acid-containing tablets in Bangladesh. We also collected information about the timing of initiation of vitamin use (before or after knowing about pregnancy), duration and frequency. To estimate nutritional intake during pregnancy, we administered a food frequency questionnaire previously validated in Bangladesh.[21] Folate status was additionally assessed in serum samples using Chemiluminescent Microparticle Immunoassay at NINS&H (ARCHITECT plus ci4100, Abbott Company, Abbott Park, IL, USA).

### Water arsenic concentration

Mothers were asked to identify the primary drinking water source they used at the time they became aware of their pregnancies. Trained staff visited these sites and collected water samples in polyethylene containers. Arsenic concentrations in water were assessed at the Bangladesh University of Engineering and Technology using graphite furnace atomic absorption spectrometry (GF-AAS) with a limit of detection (LOD) of 1 μg/L.[22] For water arsenic concentrations below the limit of detection (LOD), we assigned a value of $\text{LOD}/\sqrt{2}$. We tested one blank for every 50 water samples.

### Toenail arsenic concentration

Mother’s toenail clippings were obtained from all toenails and placed in a small coin envelope. The samples were stored and shipped to the Dartmouth Trace Element Analysis Core at room temperature. Arsenic concentrations were measured using inductively coupled plasma mass spectrometry (ICP-MS) using methods that have been previously described.[23] The instrument’s LOD was 0.01 ng/g for the first 338 samples, and 0.1 ng/g for the remaining samples. For toenail arsenic concentrations below the instrument’s LOD, we assigned a value equal to the average dilution factor of toenail samples multiplied by $(0.001*\text{LOD})/\sqrt{2}$.[20]

### Statistical analysis

To compare the data between cases and controls, we used t-tests for continuous variables, and chi-square and Fisher’s exact tests for categorical variables. We calculated Spearman correlation coefficients for the correlations of toenail and water arsenic concentrations.

We employed logistic regression models to assess the associations between arsenic concentrations and folic acid use (predictors) and case status. We modeled arsenic concentrations in two ways: 1) as a continuous variable and 2) as a binary variable: above and below 10 μg/L for water (the current World Health Organization drinking water standard for arsenic), and above and below the median for toenails. We did not use conditional models because of the uneven numbers of cases and controls. Models were adjusted for potential confounders that were chosen based on prior knowledge and using directed acyclic graphs and included mother’s age, place of birth (hospital, clinic, or home) and secondhand smoke exposure. In sensitivity analyses, we restricted the cases to only those with myelomeningocele. Data were analyzed using R (version 4.0.4).

## RESULTS

We enrolled 333 infants with initial diagnoses of spina bifida (meningocele or myelomeningocele) and 165 controls. We stopped enrolling controls and collecting water in March 2020 because of COVID-19 restrictions, and this led to an uneven number of cases and controls. Participation rates were 73% among potential families with spina bifida and 57% among potential controls. Reasons for study refusal were similar among cases and controls and primarily included concerns about giving blood. We excluded 11 cases because surgery revealed lipomeningocele and 1 case because the mother reported valproic acid use. Toenail arsenic concentrations were not available for 27 cases and 2 controls. Our final study population included 294 cases and 163 controls. Diagnoses of the controls are presented in Supplemental Table 1.

Demographic information is presented in Table 1. None of the mothers reported a diagnosis of diabetes, including gestational diabetes. Prenatal folic acid use among our study population was low, but consistent with other reports from Bangladesh;[24–26] only 16.7% of mothers of cases in our study and 22.1% of mothers of controls reported using folic acid during pregnancy. Serum folate concentrations were higher among mothers who reported folic acid use during pregnancy compared to those who did not report folic acid use (10.20 ng/ml and 7.96 ng/ml, respectively). On average, arsenic concentrations in water were lower than seen in our previous studies in Bangladesh,[19, 27] although some high arsenic concentrations were seen (maximum water arsenic concentration: 451 μg/L) (Table 2). We observed a moderate correlation between water and toenail arsenic concentrations (Spearman correlation coefficient: 0.45). Arsenic was detected in all toenail samples.

**Figure 1:**
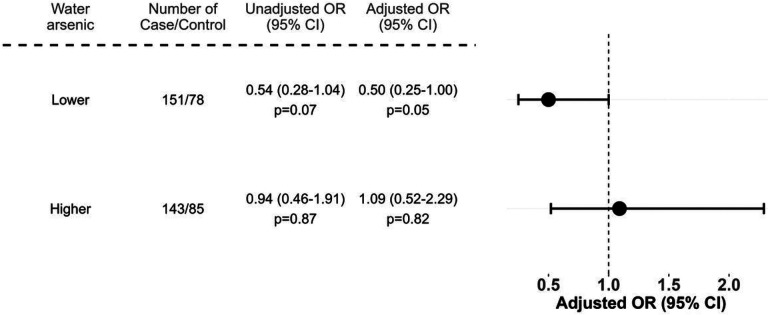
Association between prenatal folic acid use and spina bifida risk, by mother’s toenail arsenic concentration. Adjusted for mother’s age (years), place of birth (hospital, clinic, or homes), and secondhand smoke exposure. The cutoff for mother’s toenail arsenic concentrations was defined by the median of study population (0.46 μg/g toenail). Abbreviations: CI, confidence interval; OR, odds ratio.

There was no significant main effect found between water and mother’s toenail arsenic concentrations and spina bifida risk (Supplemental Table 2). However, in stratified models, we found evidence that arsenic exposure modified the effect of folic acid on spina bifida risk (Fig. 1). Among mothers with toenail arsenic concentrations below the median, folic acid use during pregnancy was associated with lower odds of having an infant with spina bifida (adjusted odds ratio [OR]: 0.50, 95% confidence interval [CI]: 0.25–1.00). Among women with toenail arsenic concentrations above the median, this protective association was not seen (adjusted OR: 1.09, 95% CI: 0.52–2.29). We found similar results after restricting the cases to those with myelomeningocele (Supplemental Fig. 1) (adjusted OR: 0.45, 95% CI: 0.21–0.94 vs. adjusted OR: 1.03, 95% CI: 0.48–2.23). When using water arsenic concentration as our measure of arsenic exposure, folic acid had a protective association at arsenic concentrations > 10 μg/L (adjusted OR: 0.18, 95% CI 0.04–0.93, p = 0.04), but this finding may be limited by the small numbers of reported folic acid users in this group (Supplemental Fig. 2).

## DISCUSSION

Our study found that arsenic modifies the effect of folic acid on spina bifida prevention in a population in Bangladesh, a country with high arsenic exposures through contaminated drinking water. Among mothers with toenail arsenic concentrations below the median, folic acid was associated with a protective effect (adjusted OR:0.50, 95% CI: 0.25–1.00), but a protective effect was not observed among mothers with toenail concentrations above the median (adjusted OR:1.09, 95% CI: 0.52–2.29). We found similar evidence of effect modification when we restricted the analysis to cases with myelomeningocele, a more severe type of spina bifida. (Supplemental Fig. 1)

Our results are consistent with recent studies conducted in high arsenic areas that suggest that environmental arsenic exposure may attenuate the protective benefit of folic acid supplements in preventing spina bifida. In a large case-control study of NTDs in Shanxi, China, researchers found that placental arsenic concentrations were associated with NTD-affected pregnancies among mothers who reported not taking folic acid supplements.[28] Our previous studies in Bangladesh found that as drinking water arsenic concentrations increased from 1 μg/L to 25 μg/L, the protective effect of folic acid use declined (OR: 0.22, 95% CI: 0.13, 0.37 to OR: 1.03, 95% CI: 0.55, 1.91).[19] These studies suggest that prenatal folic acid supplementation may be less effective in spina bifida prevention in populations with high arsenic exposure because of interactions between arsenic and folic acid.

Animal studies have consistently shown arsenic to be a potent teratogen, inducing neural tube defects in several animal models.[7–11] In addition, animal studies demonstrate that arsenic-folate interactions are important in arsenic’s teratogenicity. In *Folbp2* knockout mice, for example, mice with this specific defect in folate transport had higher rates of NTDs after arsenic exposure, and mice nullizygous for genes encoding proteins in cellular uptake of folate were more susceptible to arsenic-induced NTDs.[29] The interaction between arsenic and folate may be explained by arsenic’s interference with folate-related functions, such as the depletion of S-adenosylmethionine (SAM), a key methyl donor for pathways implicated in neural tube closure.[12] In experimental models, chicken embryos exposed to 100nM arsenate showed reduced SAM levels and higher rates of NTDs.[30] In humans, the strongest evidence for arsenic-folic acid interactions comes from trials in which folic acid has been shown to reduce blood arsenic concentrations.[31–33]

Although we found evidence of effect modification by arsenic, our study did not demonstrate a primary (or main) effect of mother’s arsenic exposure on spina bifida risk, consistent with findings from a recent systematic review assessing cohort studies that had measures of prenatal arsenic exposure and spina bifida outcomes.[13] It is possible that even higher arsenic exposures are needed to demonstrate a primary effect. A recent study from an area of Turkey with high arsenic exposures reported higher levels of arsenic in plasma samples of 100 mothers of NTD cases as compared to mothers from 70 controls. [34] Survivorship bias may also account for not finding a primary effect. We enrolled cases at time of presentation for surgical care and did not capture affected pregnancies that did not continue to birth or more severely affected infants who were not brought for care. If arsenic exposure was related to these more severe outcomes, our results could represent a downward bias towards or beyond the null.[35] Another possible explanation for why we did not find a primary effect of arsenic is that arsenic exposure by itself may be insufficient, and that the addition of other risk factors, such as inadequate folate, is a necessary contribution to disrupt neural tube closure.

The strengths of our study include the use of individual measures of exposure, including toenail arsenic measurements, which represent arsenic exposure from 5 to 18 months before collection,[36] and may better represent exposure during early pregnancy than blood, urine, or water samples collected after delivery. Previous work in Bangladesh in a large pregnancy cohort has shown high correlations between pregnant women’s toenail arsenic measurements during the first trimester and 1 month post-partum and also with measurements from their infant’s toenails at age 1 month.[27] An additional strength is classification of cases by neurosurgeons using examination, imaging and observations during surgery. It is increasingly understood that NTDs are not one disorder, but instead a wide array of morphologically distinct malformations which likely each result from a different contribution of risk factors.[37] Our study is the largest study to date to investigate spina bifida only (larger studies included all NTDs), and we were able to further restrict our analyses to only myelomeningocele in sensitivity analyses.

## CONCLUSIONS

Environmental arsenic exposure may reduce the protective effects of folic acid supplementation on spina bifida risk. Our findings raise important questions about the risk of spina bifida in arsenic-endemic areas and the effectiveness of folic acid supplements alone as the strategy to prevent spina bifida in high-arsenic areas of the world. At minimum, more surveillance for spina bifida is needed in high arsenic areas such as Bangladesh. Additional preventive measures, such as folic acid fortification of the food supply and reduction of arsenic exposure, may be needed to optimize spina bifida prevention in areas with high arsenic exposures.

## Figures and Tables

**Table 1.** Study Population

	Cases (n = 294)	Controls (n = 163)	p-value
Infant characteristics			
Boys	157 (53.4)	104 (63.8)	0.04
Firstborn	102 (34.7)	87 (53.4)	<0.001
Gestational age (week)	37.3 (2.1)	37.0 (2.3)	0.22
Age at study visit (days)	75.5 (86.6)	185.3 (96.1)	<0.001
Myelomeningocele	249 (84.7)	NA	
Level of spina bifida			
Cervical or thoracic	11 (3.7)	NA	
Lumbar	144 (49.0)	NA	
Sacral or lumbosacral	138 (46.9)	NA	
Mother’s characteristics			
Age (years)	24.7 (4.7)	24.3 (5.0)	0.39
Education level			0.11
Illiterate	3 (1.0)	2 (1.2)	
Literate	55 (18.7)	22 (13.5)	
High school or less	164 (55.8)	83 (50.9)	
College	56 (19.0)	38 (23.3)	
University	16 (5.4)	18 (11.0)	
Folic acid use during early pregnancy	49 (16.7)	36 (22.1)	0.19
Living in Dhaka	153 (52.0)	89 (54.6)	0.67
Pre-pregnancy diabetes	9 (3.1)	4 (2.5)	0.78
Diagnosis of gestational diabetes	5 (1.7)	4 (2.5)	0.73
Secondhand smoke exposure	127 (43.2)	60 (36.8)	0.22
Fever episodes during pregnancy	153 (52.0)	70 (42.9)	0.06
Place of birth			0.16
Home	79 (26.9)	41 (25.2)	
Clinics	111 (37.8)	50 (30.7)	
Hospitals	104 (35.4)	72 (44.2)	

Mean (SD) or number (%); NA = not applicable.

**Table 2.** Arsenic concentrations in toenails and drinking water

	Mean (SD)	Median	IQR	Min	Max
Mother’s toenail arsenic concentration (μg/g)					
Cases (n = 294)	1.04 (1.58)	0.45	0.28–1.05	0.07	13.77
Controls (n = 163)	1.13 (1.94)	0.47	0.30–0.96	0.09	12.30
Water arsenic concentration (μg/L)					
Cases (n = 166)	15.35 (37.28)	2.00	1.00–7.00	0.71	255.00
Controls (n = 161)	20.91 (56.08)	2.00	0.71–7.00	0.71	451.00

a. Water arsenic concentrations were below the LOD (1 μg/L) for 41 cases and 52 controls. Arsenic was detected in all toenail samples, and the LOD for toenail arsenic was 0.01 ng/g for first 338 samples tested and 0.1 ng/g for remaining samples

b. Water samples were not available for participants enrolled during the COVID-19 pandemic due to travel restrictions in Bangladesh.

Abbreviations: IQR, interquartile range; LOD, limit of detection; SD, standard deviation.

## Data Availability

Data will be made available from the corresponding author on reasonable request.

## Abbreviations

BCH: Boston Children’s Hospital
CSF: cerebrospinal fluid
CI: confidence interva
DSH: Dhaka Shishu Hospital
GF-AAS: graphite furnace atomic absorption spectrometry
ICP-MS: inductively coupled plasma mass spectrometry
LOD: limit of detection
NINS&H: National Institute of Neurosciences & Hospital
NTD: neural tube defect
OR: odds ratio
SAM: S-adenosylmethionine

## References

[1] Iskandar BJ, Finnell RH: Spina Bifida. N Engl J Med 2022, 387(5):444–450. 35921452 10.1056/NEJMra2116032

[2] Blencowe H, Kancherla V, Moorthie S, Darlison MW, Modell B: Estimates of global and regional prevalence of neural tube defects for 2015: a systematic analysis. Ann N Y Acad Sci 2018, 1414(1):31–46. 29363759 10.1111/nyas.13548

[3] De-Regil LM, Pena-Rosas JP, Fernandez-Gaxiola AC, Rayco-Solon P: Effects and safety of periconceptional oral folate supplementation for preventing birth defects. Cochrane Database Syst Rev 2015, 2015(12):CD007950. 26662928 10.1002/14651858.CD007950.pub3 PMC8783750

[4] Castillo-Lancellotti C, Tur JA, Uauy R: Impact of folic acid fortification of flour on neural tube defects: a systematic review. Public Health Nutr 2013, 16(5):901–911. 22850218 10.1017/S1368980012003576 PMC10271422

[5] Atta CA, Fiest KM, Frolkis AD, Jette N, Pringsheim T, St Germaine-Smith C, Rajapakse T, Kaplan GG, Metcalfe A: Global Birth Prevalence of Spina Bifida by Folic Acid Fortification Status: A Systematic Review and Meta-Analysis. Am J Public Health 2016, 106(1):e24–34. 26562127 10.2105/AJPH.2015.302902 PMC4695937

[6] Cordero A, Mulinare J, Berry RJ, Boyle C, Dietz W, Johnston R Jr., Popovic T: CDC Grand Rounds: Additional Opportunities to Prevent Neural Tube Defects with Folic Acid Fortification. Morbidity and Mortality Weekly Report 2010, 59(31):980–984. 20703205

[7] Wlodarczyk BJ, Bennett GD, Calvin JA, Finnell RH: Arsenic-induced neural tube defects in mice: alterations in cell cycle gene expression. Reprod Toxicol 1996, 10(6):447–454. 8946558 10.1016/s0890-6238(96)00131-1

[8] Hill DS, Wlodarczyk BJ, Finnell RH: Reproductive consequences of oral arsenate exposure during pregnancy in a mouse model. Birth Defects Research Part B: Developmental and Reproductive Toxicology 2008, 83(1):40–47. 18186108 10.1002/bdrb.20142

[9] Hood RD, Bishop SL: Teratogenic effects of sodium arsenate in mice. Arch Environ Health 1972, 24(1):62–65. 5009633 10.1080/00039896.1972.10666051

[10] Beaudoin AR: Teratogenicity of sodium arsenate in rats. Teratology 1974, 10(2):153–157. 4428424 10.1002/tera.1420100211

[11] Carpenter SJ: Developmental analysis of cephalic axial dysraphic disorders in arsenic-treated hamster embryos. Anat Embryol 1987, 176(3):345–365. 10.1007/BF00310189 3631535

[12] Abuawad A, Bozack AK, Saxena R, Gamble MV: Nutrition, one-carbon metabolism and arsenic methylation. Toxicology 2021, 457:152803. 33905762 10.1016/j.tox.2021.152803 PMC8349595

[13] Eaves LA, Choi G, Hall E, Sille FCM, Fry RC, Buckley JP, Keil AP: Prenatal Exposure to Toxic Metals and Neural Tube Defects: A Systematic Review of the Epidemiologic Evidence. Environ Health Perspect 2023, 131(8):86002. 37647124 10.1289/EHP11872 PMC10467818

[14] Chakraborti D, Rahman MM, Mukherjee A, Alauddin M, Hassan M, Dutta RN, Pati S, Mukherjee SC, Roy S, Quamruzzman Q : Groundwater arsenic contamination in Bangladesh-21 Years of research. J Trace Elem Med Biol 2015, 31:237–248. 25660323 10.1016/j.jtemb.2015.01.003

[15] Smith AH, Lingas EO, Rahman M: Contamination of drinking-water by arsenic in Bangladesh: a public health emergency. Bull World Health Organ 2000, 78(9):1093–1103. 11019458 PMC2560840

[16] Bangladesh Bureau of Statistics (BBS) and UNICEF Bangladesh: Progotir Pathey, Bangladesh Multiple Indicator Cluster Survey 2019, Survey Findings Report. In.: Dhaka, Bangladesh: Bangladesh Bureau of Statistics (BBS). 2019.

[17] Shuma ML, Halder S, Datta BK: Epidemiology of congenital anomalies among the children born in different hospitals under Sylhet Division in Bangladesh - a retrospective study. Dhaka Univ J PharSci 2015, 14(2):225–230.

[18] Dey AC, Shahidullah M, Mannan MA, Noor MK, Saha L, Rahman SA: Maternal and neonatal serum zinc level and its relationship with neural tube defects. J Health Popul Nutr 2010, 28(4):343–350. 20824977 10.3329/jhpn.v28i4.6040 PMC2965325

[19] Mazumdar M, Ibne Hasan MO, Hamid R, Valeri L, Paul L, Selhub J, Rodrigues EG, Silva F, Mia S, Mostofa MG : Arsenic is associated with reduced effect of folic acid in myelomeningocele prevention: a case control study in Bangladesh. Environ Health 2015, 14(1):34. 25885259 10.1186/s12940-015-0020-0 PMC4404044

[20] Tindula G, Mukherjee SK, Ekramullah SM, Arman DM, Biswas SK, Islam J, Obrycki JF, Christiani DC, Liang L, Warf BC : Parental metal exposures as potential risk factors for spina bifida in Bangladesh. Environ Int 2021, 157:106800. 34358915 10.1016/j.envint.2021.106800 PMC9008873

[21] Chen Y, Ahsan H, Parvez F, Howe GR: Validity of a food-frequency questionnaire for a large prospective cohort study in Bangladesh. Br J Nutr 2004, 92(5):851–859. 15533275 10.1079/bjn20041277

[22] Frisbie SH, Mitchell EJ, Yusuf AZ, Siddiq MY, Sanchez RE, Ortega R, Maynard DM, Sarkar B: The development and use of an innovative laboratory method for measuring arsenic in drinking water from western Bangladesh. Environ Health Perspect 2005, 113(9):1196–1204. 16140627 10.1289/ehp.7974 PMC1280401

[23] Chen KL, Amarasiriwardena CJ, Christiani DC: Determination of total arsenic concentrations in nails by inductively coupled plasma mass spectrometry. Biol Trace Elem Res 1999, 67(2):109–125. 10073418 10.1007/BF02784067

[24] Kancherla V, Ibne Hasan MOS, Hamid R, Paul L, Selhub J, Oakley G, Quamruzzaman Q, Mazumdar M: Prenatal folic acid use associated with decreased risk of myelomeningocele: A case-control study offers further support for folic acid fortification in Bangladesh. PLoS One 2017, 12(11):e0188726. 29190654 10.1371/journal.pone.0188726 PMC5708673

[25] Khambalia A, O’Connor DL, Zlotkin S: Periconceptional iron and folate status is inadequate among married, nulliparous women in rural Bangladesh. J Nutr 2009, 139(6):1179–1184. 19403710 10.3945/jn.108.101022

[26] Alam N, Roy SK, Ahmed T, Ahmed AM: Nutritional status, dietary intake, and relevant knowledge of adolescent girls in rural Bangladesh. J Health Popul Nutr 2010, 28(1):86–94. 20214090 10.3329/jhpn.v28i1.4527 PMC2975850

[27] Rodrigues EG, Kile M, Dobson C, Amarasiriwardena C, Quamruzzaman Q, Rahman M, Golam M, Christiani DC: Maternal-infant biomarkers of prenatal exposure to arsenic and manganese. J Expo Sci Environ Epidemiol 2015, 25(6):639–648. 26306926 10.1038/jes.2015.45 PMC4770909

[28] Pi X, Wang C, Yin S, Jin L, Li Z, Wang L, Liu J, Zhang Y, Ren A: Arsenic Exposure, Periconceptional Folic Acid Supplementation, and the Risk for Neural Tube Defects: A Case–Control Study. Exposure and Health 2022, 15(1):245–254.

[29] Wlodarczyk BJ, Cabrera RM, Hill DS, Bozinov D, Zhu H, Finnell RH: Arsenic-induced gene expression changes in the neural tube of folate transport defective mouse embryos. Neurotoxicology 2006, 27(4):547–557. 16620997 10.1016/j.neuro.2006.02.005

[30] Han ZJ, Song G, Cui Y, Xia HF, Ma X: Oxidative stress is implicated in arsenic-induced neural tube defects in chick embryos. Int J Dev Neurosci 2011, 29(7):673–680. 21723934 10.1016/j.ijdevneu.2011.06.006

[31] Gamble MV, Liu X, Ahsan H, Pilsner JR, Ilievski V, Slavkovich V, Parvez F, Chen Y, Levy D, Factor-Litvak P : Folate and arsenic metabolism: a double-blind, placebo-controlled folic acid-supplementation trial in Bangladesh. Am J Clin Nutr 2006, 84(5):1093–1101. 17093162 10.1093/ajcn/84.5.1093 PMC2046214

[32] Gamble MV, Liu X, Slavkovich V, Pilsner JR, Ilievski V, Factor-Litvak P, Levy D, Alam S, Islam M, Parvez F : Folic acid supplementation lowers blood arsenic. Am J Clin Nutr 2007, 86(4):1202–1209. 17921403 10.1093/ajcn/86.4.1202 PMC2042963

[33] Peters BA, Hall MN, Liu X, Parvez F, Sanchez TR, van Geen A, Mey JL, Siddique AB, Shahriar H, Uddin MN : Folic Acid and Creatine as Therapeutic Approaches to Lower Blood Arsenic: A Randomized Controlled Trial. Environ Health Perspect 2015, 123(12):1294–1301. 25978852 10.1289/ehp.1409396 PMC4671237

[34] Demir N, Başaranoğlu M, Huyut Z, Değer İ, Karaman K, Şekeroğlu MR, Tuncer O: The relationship between mother and infant plasma trace element and heavy metal levels and the risk of neural tube defect in infants. The Journal of Maternal-Fetal & Neonatal Medicine 2019, 32(9):1433–1440. 29199526 10.1080/14767058.2017.1408064

[35] Leung M, Kioumourtzoglou MA, Raz R, Weisskopf MG: Bias due to Selection on Live Births in Studies of Environmental Exposures during Pregnancy: A Simulation Study. Environ Health Perspect 2021, 129(4):47001. 33793300 10.1289/EHP7961 PMC8043129

[36] Signes-Pastor AJ, Gutierrez-Gonzalez E, Garcia-Villarino M, Rodriguez-Cabrera FD, Lopez-Moreno JJ, Varea-Jimenez E, Pastor-Barriuso R, Pollan M, Navas-Acien A, Perez-Gomez B : Toenails as a biomarker of exposure to arsenic: A review. Environ Res 2021, 195:110286. 33075355 10.1016/j.envres.2020.110286 PMC7987585

[37] Wallingford JB, Niswander LA, Shaw GM, Finnell RH: The continuing challenge of understanding, preventing, and treating neural tube defects. Science 2013, 339(6123):1222002. 23449594 10.1126/science.1222002 PMC3677196

